# The Small Fibrous Prostate

**Published:** 1915-05

**Authors:** A. L. Fowler

**Affiliations:** Atlanta, Ga.; Professor of Genito-Urinary Surgery in the Atlanta Medical College; Genito-Urinary Surgeon to the Grady (Municipal) Hospital; Genito-Urinary Surgeon to St. Joseph Infirmary; Member American Urological Association; Member American Medical Association, Etc.


### THE SMALL FIBROUS PROSTATE

By A. L. Fowler, M. D., Atlanta, Ga.
Professor of Genito-Urinary Surgery in the Atlanta Medical College; Genito-Urinary Surgeon to the Grady (Municipal) Hospital; Genito-Urinary Surgeon to St. Joseph Infirmary; Member American Urological Association; Member American Medical Association, Etc.

The small fibrous prostate properly belongs under the heading of enlarged, or hypertrophied, prostate because it can operate to cause quite as much, if not more, residual urine than the big prostates.

Because this type—the small fiibrous prostate—is so frequently overlooked, not only by the physician with his palpating finger in the rectum, but also by the general surgeon after he has opened the bladder supra-pubically, is my excuse for reading this paper before the association.

The cause of these small, hard, fibrous prostates is infection; but not necessarily venereal, since those of us who have seen many of them know full well that some of our cases have had no urethral infection, nor has there been a history of anything like an acute prostatitis. Of course, some of the cases will give a history of gonnorrhœa; indeed, about one in three of my cases gave such a history. But, at that, is it not likely that the previous gonnorrhœa in no way whatsoever operated to cause this fibrosis? The presumption is, naturally, that in those cases giving a history of gonorrhœa this organism caused the disease. In the others such presumptions are not presumable, and some other agent is the etiological factor.

Ungratified sexual desire, excessive venery, coitus reservatus, and so forth, may lower the resisting powers of the gland so that bacteria find little difficulty in advancing to the attack, and with a low grade of infection causing the mischief the process might easily progress slowly and surely, but quite insiduously. The disease must, in many cases, be due to hæmatogenous or lymphogenous infection of some kind; but it certainly is not always venereal. I have seen the disease in men who knew not what venereal diseases were; in men who lived in the wilds of the mountains, some of whom had never seen a train until they came under my medical care at the United States Penitentiary Hospital, they having been arrested for illicit distilling far from civilization.

In examining the prostate by rectum one should not, unless he be an expert, give too much weight to apparent changes as conveyed to the palpating finger. Some prostates that are big, bulge bladder-ward and cannot be felt as enlarged when palpated per rectum, yet they occasion a great amount of residual urine. In such cases a catheter passed after the patient urinates, or a retrograde (not an ordinary) cystoscope will point the way.

DIAGNOSIS.

The diagnosis of the small fibrous prostate can be most puzzling, particularly if the examiner proceeds with the idea that a prostate must be enlarged, per rectum, in order to cause prostatic obstruction. The recognition of this type becomes easier if it be remembered that every man experiencing difficulty in urination possibly has a small fibrous prostate. Usually such a gland is felt as a small, but clearly defined fibrous-feeling body, with a well marked sulcus. Occasionally, scarcely any gland can be mapped out with the finger.

In these cases there is a marked resistance to a soft rubber catheter at the bladder outlet; and the passage of a steel sound is accompanied by a gripping of the instrument as it engages the contracted bladder-neck. The difficulty in instrumentation is due to the formation of a small fibrous ring of prostatic tissue that has shrunken around the beginning of the prostatic urethra.

After a small fibrous prostate has existed for a sufficient length of time there will be a variable amount of residual urine. A small fibrous prostate will occasionally cause the presence of more residual urine than a greatly enlarged prostate. If we can rule out stricture, diverticulum of the bladder and lesion of the spinal cord it is reasonable to assume that a residual urine owes its presence to some abnormality of the prostate.

Rertograde cystoscopy, in doubtful cases, will disclose trabeculated bladder walls (from its efforts at emptying itself), and a slight elevation of the floor of the vesico-urethral opening above the trigone. If the instrument be rotated a small, red ring may be observed hard around the circumference of the shaft of the retrograde cystoscope.

TREATMENT.

The treatment is to remove as much of this fibrous tissue as possible and, at the outset, let me say this is not easy. I have operated on these cases both by the low and the high routes, and rather favor the high route because of

1. The advantage of sight with bladder illumination.

2. Clear view of entire bladder-cavity and mucosa before and after operation.

3. Clear view of the gland before operation; good view during operation and afterwards.

4. Good view of membranous tags, after attacking the prostate, which should be removed to avoid subsequent mischief.

After removal of all the fibrous tissue possible there will generally remain a small, tight fibrous outlet and which is very unlike the spacious outlet seen after removal of an enlarged prostate. The best results are obtained by splitting this remaining ring upon its floor, or upon its floor and to one side. This latter procedure is very important and if it be overlooked the operation may be a failure.

The subsequent treatment of the bladder is very like that for enlarged prostate, the routine treatment of which is unnecessary to detail in this paper.

I have brought the subject of small fibrous prostate before the association because it is quite likely to be overlooked not only by the general practitioner, but by the general surgeon as well. During the past few years I have operated on three cases that had been previously drained suprapubically by good surgeons. These cases were temporarily benefitted, but as soon as the suprapubic incision healed all the former symptoms returned. This points out how a very good surgeon can easily fail to recognize the condition, notwithstanding the bladder is open to inspection, via the space of Retzius, before him; further, he fails to make use of the palpating finger when he can actually introduce it into the bladder cavity. This being true, is it any wonder that the condition is overlooked so frequently with an unskilled finger in the patient's rectum?

Bibliography: Chute, Legueu.

929 Candler Building.

